# Up-regulation of p21 and TNF-α is mediated in lycorine-induced death of HL-60 cells

**DOI:** 10.1186/1475-2867-10-25

**Published:** 2010-08-04

**Authors:** Jing Liu, Ji-liang Hu, Bi-Wei Shi, Yan He, Wei-Xin Hu

**Affiliations:** 1 Molecular Biology Research Center, School of Biological Science and Technology, Central South University, 110 Xiangya Road, Changsha, Hunan 410078, People's Republic of China

## Abstract

**Background:**

Leukemia is one of the most life-threatening cancers today, and acute promyelogenous leukemia (APL) is a common type of leukemia. Many natural compounds have already been found to exhibit significant anti-tumor effects. Lycorine, a natural alkaloid extracted from Amaryllidaceae, exhibited anti-leukemia effects in vitro and in vivo. The survival rate of HL-60 cells exposed to lycorine was decreased, cell growth was slowed down, and cell regeneration potential was inhibited. HL-60 cells exhibited typical apoptotic characteristic. Lycorine can suppress leukemia growth and reduce cell survival and inducing apoptosis of tumor cells. The purpose of this work is to elucidate the mechanism by which lycorine induces APL cells.

**Results:**

When HL-60 cells were treated with different concentration of lycorine, the expression of p21 and TNF-α was up-regulated in a concentration-dependent manner as shown by real-time quantitative reverse transcriptase-polymerase chain reaction and Western blotting. Lycorine also down-regulated p21-related gene expression, including Cdc2, Cyclin B, Cdk2 and Cyclin E, promoted Bid truncation, decreased IκB phosphorylation and blocked NF-κB nuclear import. Cytochrome c was released from mitochondria as observed with confocal laser microscopy.

**Conclusions:**

The TNF-α signal transduction pathway and p21-mediated cell-cycle inhibition were involved in the apoptosis of HL-60 cells induced by lycorine. These results contribute to the development of new lycorine-based anti-leukemia drugs.

## Background

A tumor is a disease with two defining characteristics: a proliferation disorder and an apoptosis obstacle. The inhibition of proliferation and the induction of apoptosis are regulated by a network of signaling pathways and transcription factors, which may represent potential targets for rational tumor therapy [1,2]. Apoptotic events are regulated by the interplay of proapoptotic and antiapoptotic proteins. The apoptotic pathways include two major signaling pathways: the death receptor-induced pathway and the mitochondria-apoptosome-mediated pathway. Elements of the death receptor pathway include cell death ligands and their receptors, such as tumor necrosis factor (TNF) and tumor necrosis factor-related apoptosis-inducing ligand (TRAIL) receptor, and downstream molecules, such as caspase 8. The major components of the mitochondrial pathway include apoptotic stimuli, mitochondria, the apoptosome, and key effector caspases [1]. Crosstalk between these two apoptotic pathways is mediated through the truncation of the BH3-interacting death domain (Bid) protein. Inhibitors of apoptosis proteins include the X-linked inhibitor of apoptosis protein (XIAP), the cellular inhibitor of apoptosis protein (cIAP), survivin, and the phosphatidyl inositol 3 kinase/serine/threoninespecific protein kinase/nuclear factor-kappa B (PI3K/AKT/NF-κB) pathway. Recent studies had focused on inducing cancer cell apoptosis by targeting the core components of the apoptosis-related signaling pathway and had produced promising results [3,4]. Cancer is also a disease of the cell cycle, a series of events that a eukaryotic cell must undergo to result in its replication. The cyclin-dependent kinase inhibitor p21 plays a key role in cell-cycle regulation. Gartel and Tyner [5] showed that p21-dependent cell cycle arrest occurs at the G2/M phase. The key regulator of the G2/M phase is the cell division cycle 2 (Cdc2)/Cyclin B, the activity of which can be regulated by p21 [6]. Furthermore, p21 possesses proapoptotic functions in some systems. Overexpression of p21 increases the susceptibility of glioma cell lines [7] and several p53-deficient human cell lines [8] to chemotherapeutic agent-induced apoptosis. These studies had revealed a correlation between the expression level of p21 and patient survival [9].

Alkaloids have been found to exhibit effective anti-cancer activities with multiple mechanisms. For example, camptothecin and its analogs exhibited strong anti-cancer activities by inhibiting the DNA-uncoiling function of topoisomerase I [10]. Shikonin induced the apoptosis of Bcr/Abl-positive chronic myelogenous leukemia (CML) cells through a reactive oxygen species/c-Jun N-terminal kinase (ROS/JNK)-mediated process [11]. Homoharringtonine promoted apoptosis in K562 cells [12] and more than 90% of the leukaemic stem cells were killed after treatment with homoharringtonine in vitro [13]. Leukemia is one of the most life-threatening hematological malignant cancers. Because of its potential sensitivity to chemical reagents [14,15], scientists are attempting to discover new specific and effective chemical drugs to fight this disease [16]. Lycorine, an alkaloid extracted from Amaryllidaceae, has multiple pharmacological functions, such as anti-virus effects, anti-tumour effects, and emetic action [17,18]. In our previous work, we found that lycorine decreased the survival rate and inducing apoptosis in leukemia and multiple myeloma cell lines[19,20], and the mechanisms of induced apoptosis have mediated by stimulating the caspase pathway and increasing the Bax:Bcl-2 ratio through the down-regulation of Bcl-2 expression. In addition, lycorine induced apoptosis in human leukemia cells via the mitochondria pathway and caused a rapid turnover of myeloid cell leukemia 1 (Mcl-1) protein, which occurred before caspase activation [21]. Lycorine exerted its in vitro antitumor activity through cytostatic rather than cytotoxic effects. Also, lycorine provided significant therapeutic benefit in mice bearing brain grafts of the B16F10 melanoma model at nontoxic doses [22]. Lycorine exhibited in vivo anti-tumor activity when tested in SCID mice model with human acute promyelocytic leukemia (APL) cells and was a useful therapy against [23]. The human leukemia (Jurkat) cells were investigated with the treatment of synthetic and natural lycorane alkaloids. The results showed that a free ring-C 1,2-diol in the lycorine series (lycorine and pseudolycorine) was required for potent apoptosis-inducing activity [24]. The aim of this study was to investigate the molecular mechanism underlying lycorine-induced death of HL-60 cells and provide a mechanistic framework for further exploring the use of lycorine as a novel anti-leukemia agent.

## Results

### Up-regulation of p21 expression and down-regulation of its regulated genes

**Figure 1:**
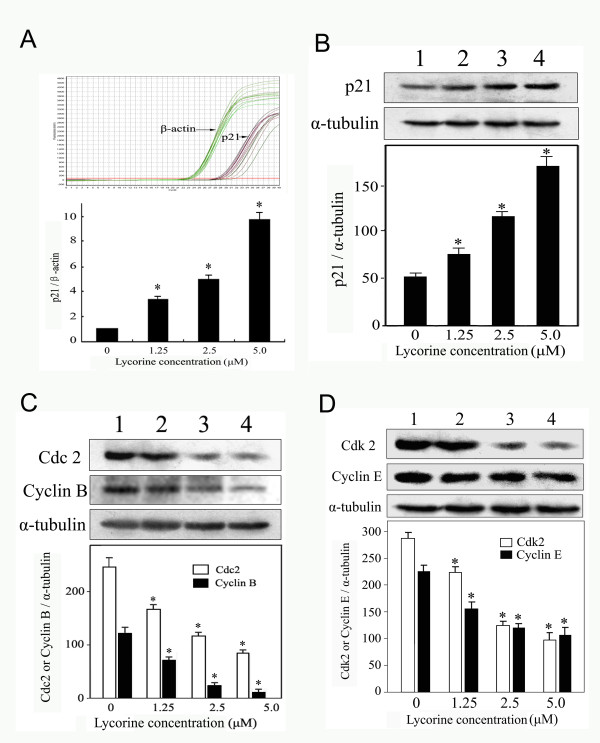
**Effects of lycorine on expression of p21 and downstream genes regulated by p21 in HL-60 cells**. (A) Expression levels of p21 in HL-60 cells were analysed by real-time quantitative RT-PCR. Expression levels were standardised using expression of the housekeeping gene β-actin. (B) Effects of lycorine on expression of p21 protein, (C) Cdc2 and Cyclin B proteins, (D) Cdk2 and Cyclin E proteins. α-tubulin was used for normalisation and verification of the protein loading in B, C, and D in Western blotting. Lanes 1-4, HL-60 cells were treated with 0, 1.25, 2.5, and 5.0 μM lycorine, respectively. Results were presented as mean ± S.D. (n = 3, three independent experiments). Asterisk symbol (*) indicates significant difference (p < 0.05) compared with the control group.

Up-regulation of the p21 gene was detected with real-time quantitative RT-PCR when HL-60 cells were treated with 5.0 μM lycorine (Fig. 1A). Western blotting (Fig. 1A, B) also showed that lycorine significantly up-regulate p21 mRNA and protein in a concentration-dependent manner. Because p21 regulated the activity of Cdc2-Cyclin B and Cdk2-Cyclin E, and the expression of Cdc2, Cyclin B, Cdk2, and Cyclin E could be detected by Western blotting. Results showed that these proteins were significantly down-regulated in a concentration-dependent manner after treatment with lycorine (Fig. 1C and 1D).

### Up-regulation of TNF-α and increase of truncated Bid (tBid)

**Figure 2:**
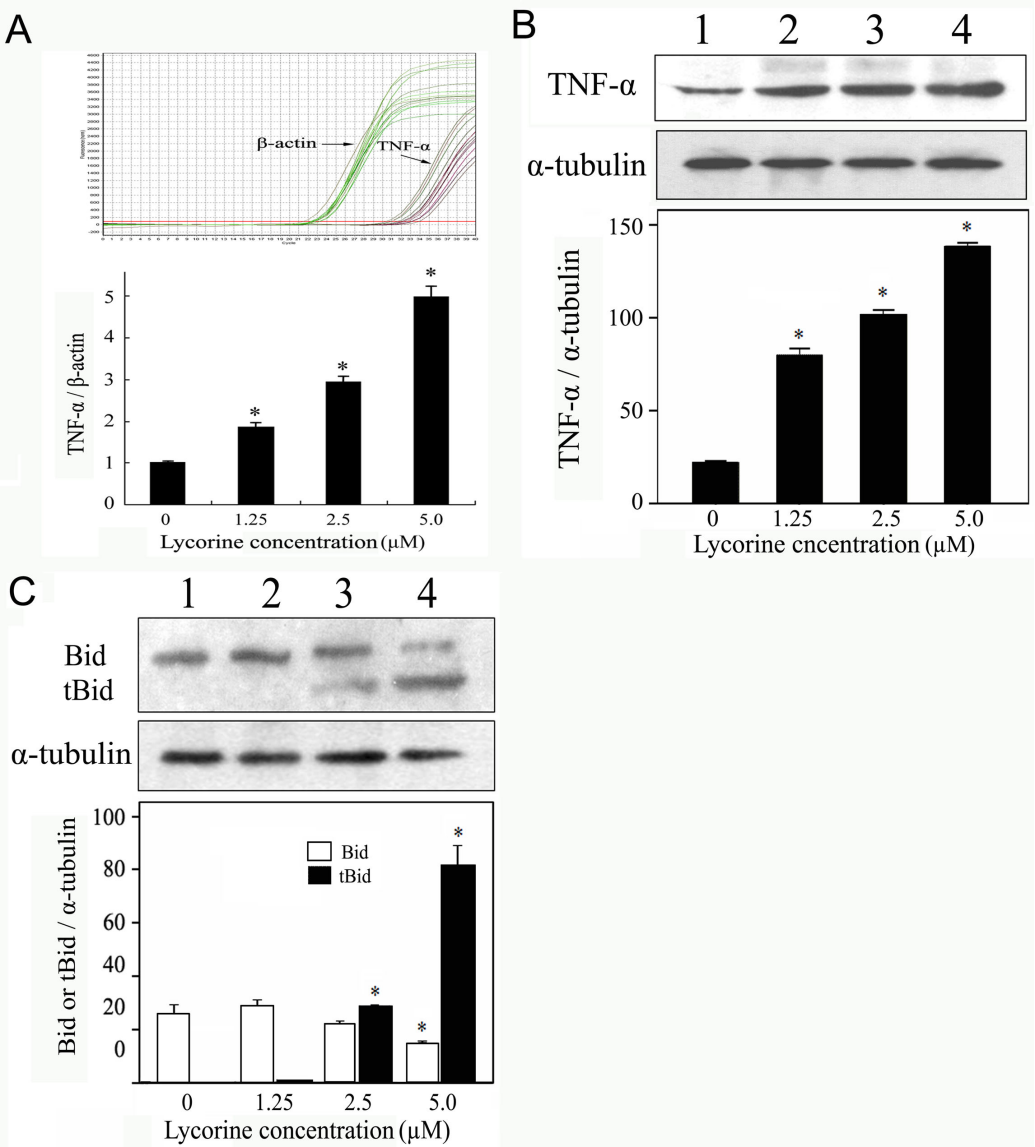
**Effects of lycorine on expression of TNF-α and truncation of Bid in HL-60 cells**. (A) Expression levels of TNF-α in HL-60 cells were analysed by real-time quantitative RT-PCR. Expression levels were standardised using expression of the housekeeping gene β-actin. (B) Effect of lycorine on expression of TNF-α protein, (C) Bid and truncation of Bid. α-tubulin was used for normalisation and verification of the protein loading in B and C in Western blotting. Lanes 1-4, HL-60 cells were treated with 0, 1.25, 2.5, and 5.0 μM lycorine, respectively. Results were presented as mean ± S.D. (n = 3, three independent experiments). Asterisk symbol (*) indicates significant difference (p < 0.05) compared with the control group.

The results of real-time RT-PCR and Western blotting (Fig. 2A and 2B) revealed that the expression of TNF-α was significantly up-regulated in a concentration-dependent manner in HL-60 cells treated with 5.0 μM lycorine for 24 h. Furthermore, Western blotting showed that the expression level of Bid protein was decreased when the lycorine concentration reached 5.0 μM and that the expression level of truncated Bid (tBid) was significantly increased in a concentration-dependent manner (Fig. 2C).

### Effect of lycorine on cytochrome c release

**Figure 3:**
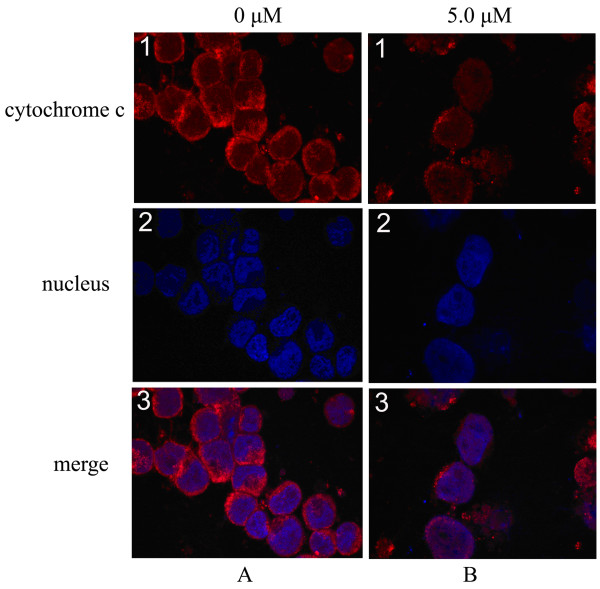
**Effect of lycorine on cytochrome c release**. Cells were fixed and labelled for cytochrome c (red) and DNA (blue). (A) HL-60 cells in control group. (B) Cells were treated with 5.0 μM lycorine for 24 h. Cell nuclei were observed by DNA staining with DAPI (A2, B2). The staining pattern of cytochrome c became diffusive in most cells (B1), consistent with a translocation of cytochrome c into the cytosol and nucleus (B3), whereas cytochrome c displayed a dotted pattern in untreated cells, consistent with its location within mitochondria (A1, A3). Images were obtained with a confocal microscope (×400).

tBid may promote the release of cytochrome c and subsequent activation of caspase 9 and caspase 3, which in turn leads to cell apoptosis. We examined the subcellular localisation of cytochrome c to determine whether cytochrome c was released from the mitochondria into the cytosol when lycorine initiates the apoptosis pathway. Immunofluorescence of cytochrome c was visualized with a confocal laser microscope. In both groups of cells (the experimental group and the control group), the nucleus appeared blue when the DNA was stained with Hoechst 33258 (Fig. 3A2 , 3B2) and the cytochrome c appeared red when stained with cyanine 3 (Cy3) fluorochrome (Fig. 3A1, B1). After the cells were treated with 5 μM lycorine for 24 h, the staining patterns of cytochrome c became diffuse and blurred (Fig. 3B1) in contrast to the compact, plaque-like appearance of cytochrome c in the control group (Fig. 3A1), indicating the translocation of cytochrome c from the mitochondria into the cytosol in the treated cells. We also found that some red dye had entered the nucleus and mixed with the blue stain to yield a purple-stained nucleus (Fig. 3B3).

### Inhibition of IκB phosphorylation and blockade of NF-κB nuclear import

**Figure 4:**
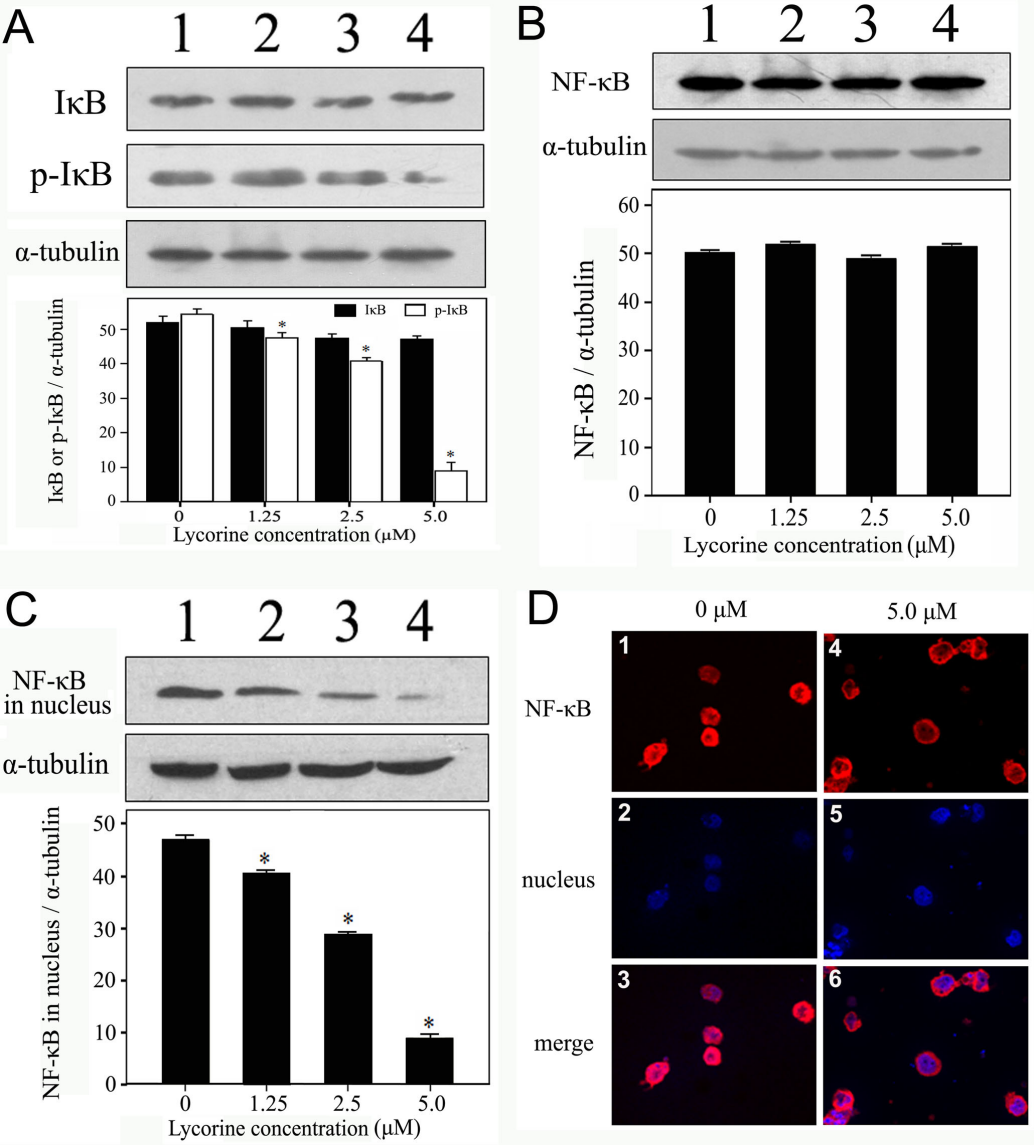
**Effects of lycorine on phosphorylation of IκB and nuclear import of NF-κB**. (A) Effect of lycorine on expression of IκB and phosphorylation of IκB protein. (B) Effect of lycorine on expression of NF-κB protein and (C) distribution of NF-κB protein in nucleus. α-tubulin was used for normalisation and verification of the protein loading in A, B, and C in Western blotting. Lanes 1-4: HL-60 cells were treated with 0, 1.25, 2.5, and 5.0 μM lycorine, respectively. (D) Subcellular distribution of NF-κB detected by immunofluorescence staining. HL-60 cells were treated with 0 μM (D1, D2, D3) and 5.0 μM lycorine (D4, D5, D6), respectively. Cells were labeled for NF-κB (red) and DNA (blue). Cell nuclei were stained by DAPI (D2, D5). NF-κB was mainly distributed in the cytoplasm (D4, D6) in the HL-60 cells treated with lycorine, whereas NF-κB was mainly distributed in the nucleus in untreated cells (D1, D3). Results were presented as mean ± S.D. (n = 3, three independent experiments). Asterisk symbol (*) indicates significant difference (p < 0.05) compared with the control group.

The expression levels of IκB and NF-κB were not obviously changed in HL-60 cells treated with lycorine; however, the phosphorylation level of IκB was significantly decreased when the concentration of lycorine reached 5.0 μM (Fig. 4A and 4B). To investigate the localization and nuclear import of NF-κB, nuclear proteins were extracted from HL-60 cells treated with different concentrations of lycorine and subsequent Western blotting was performed. The results showed that the expression of NF-κB protein in the nucleus decreased significantly in HL-60 cells (Fig. 4C). Immunocytochemistry results showed that lycorine significantly blocked the nuclear import of NF-κB protein. A comparison of treated and untreated cells revealed that NF-κB was mainly distributed in the cytoplasm of the HL-60 cells treated with lycorine (Fig. 4D4, D6), whereas NF-κB was mainly distributed in the nucleus of untreated HL-60 cells (Fig. 4D1, D3).

## Discussion

Studies reported changes in gene expression of p21 can prohibit cell proliferation and induce cell apoptosis [25,26]. p21, a key member of the Cip/Kip family and a cyclin-dependent kinase inhibitor, can bind to and directly inhibit the activity of Cyclin E-Cdk2 and Cyclin B-Cdc2 [6,27,28]. Overexpression of p21 can lead to cell cycle progression at G1, G2/M or S-phase arrest. The expression of p21 is tightly controlled by the tumour suppressor protein p53, through which this protein mediates the p53-dependent cell-cycle arrest at G1 phase in response to a variety of stress stimuli [29]. We discovered that p21 was up-regulated in HL-60 cells treated with lycorine and that both Cyclin B-Cdc2 and Cyclin E-Cdk2 were significantly down-regulated following lycorine treatment. Our findings suggested that lycorine regulated the expression of p21, and then influenced the expression of down-stream genes Cyclin B-Cdc2 and Cyclin E-Cdk2, resulting in cell cycle arrest in HL-60 cells. Because p21 has been shown to possess proapoptotic functions in p53-deficient systems [5,8], we hypothesized that p21 also functions as a proapoptotic molecule in the HL-60 cell line, a p53-deficient leukemia cell line. Our recent research results showed that p21 was also up-regulated after lycorine treated in K562 cell line (data not shown), a p53-indeficient leukemia cell line. Thus it implies that the up-regulation of p21 go through p53-independent pathways, and it is possible that p21 acts as a direct target after lycorine treatment. In the further, it is very necessary to silence p21 to confirm whether p21 is the direct target of lycorine in HL-60 cells and figure out the upstream factors by which up-regulate p21.

Our results showed that the expression of TNF-α was up-regulated in HL-60 cells treated with lycorine. It means the TNF-α signaling pathway played an important role in apoptosis induced by lycorine. Furthermore, we discovered that lycorine promoted the truncation of Bid protein, which can promote the release of cytochrome c from mitochondria [30] and, ultimately, cell apoptosis. We previously showed that lycorine promoted the release of cytochrome c and increased the activities of caspases 3, 8, and 9 in HL-60 cells and KM3 cells. Thus, we conclude that both the death-receptor-induced pathway and the mitochondria-apoptosome-mediated pathway were involved in the lycorine-induced apoptosis in HL-60 cells and that the Bid protein mediated the crosstalk between these two apoptotic pathways. p21 may also play an key role in apoptosis induced by activation of members of the TNF death receptor family [31].

NF-κB, an important transcription factor, regulates the transcription of many genes related to immune and inflammatory responses, cell differentiation, and apoptosis when transported into the cell nucleus [32]. An NF-κB-binding region was found within the upstream promoter region of several members of the Bcl2 family [33]. When NF-κB combined with IκB in the cytoplasm, NF-κB lost its ability to regulate transcription. However, when IκB was phosphorylated and released from the IκB-NF-κB complex, NF-κB regained this ability [34]. In this study, we found that lycorine decreased the phosphorylation of IκB in HL-60 cells, thus blocking the nuclear import of NF-κB. This finding indicates that the NF-κB signaling pathway was inhibited when lycorine induced HL-60 cell apoptosis.

From our results, we can deduce that cell apoptosis induced by lycorine is a very complex process involving the interaction of multiple signal transduction pathways and it deserves further study.

## Conclusions

The TNF-α signalling transduction pathway and p21-mediated cell-cycle prohibition were involved in lycorine-induced death of HL-60 cells. These results may help in the development of new anti-leukemia drugs.

## Methods

### Cell culture and lycorine treatment

The human acute promyelocytic leukemia (APL) cell line HL-60 (ATCC Number: CCL-240) was cultured in RPMI-1640 medium containing 10% fetal bovine serum (FBS), 100 U/ml penicillin, and 100 mg/L streptomycin at 37°C with 5% CO_2 _and saturated humidity. When the cell density reached 5 × 10^5 ^to 1 × 10^6^/ml, the cells of the experimental group were treated with lycorine, untreated cells represented the control.

### RNA extraction and real-time RT-PCR analyses

Total cellular RNA was extracted with Trizol reagent (Invitrogen, Carlsbad, CA, USA) from cells treated with various concentrations of lycorine (0, 1.25, 2.5, 5.0 μM) for 24 hours. Real-time quantitative reverse transcriptase-polymerase chain reaction (RT-PCR) was performed to detect the gene expression of p21 and TNF-α, and β-actin was used as the internal standard reference. The primer sequences, which were designed using Primer 5 software in accordance with the GenBank sequence, are listed in Table 1. 1.0 μg of total RNA of each group and 0.5 μg of oligo (dT)_16 _were added to an eppendorf tube and incubated at 65°C for 5 min. Then the reaction system volume was adjusted to 20 μl by adding 4.0 μl of 5× MMLV RT buffer, 20 units of RNasin, 1.0 μl of dNTPs (10 mmol), and 10 units of MMLV reverse transcriptase; incubated at 42°C for 60 min; and kept at 75°C for 5 min to terminate the reaction. The PCR reaction system (20 μl) was prepared by mixing 10 μl of SYBR premix *Ex Taq *DNA Polymerase (Takara), 2 μl of reverse transcription products, and 10 pmol mixtures of the upstream and downstream primers and distilled water. The PCR mixture was denatured at 95°C for 10 s, and amplification was run at 95°C for 15 s, 56°C for 20 s, and 68°C for 20 s for 40 cycles in the Mastercycler Realplex2 (Eppendorf).

**Table 1:** The primer sequences and their lengths

Primers	Sequences	Product length
p21 Forward	5'-AAGACCATGTGGACCTGTCACTGT-3'	155 bp for p21
p21 Reverse	5'-GAAGATCAGCCGGCGTTTG -3'	
TNF-α Forward	5'-CCCTCAGCAAGGACAGCAGA -3'	139 bp for TNF-α
TNF-α Reverse	5'-AGCCGTGGGTCAGTATGTGAGA -3'	
β-actin Forward	5'-TGACGGTCAGGTCATCACTATCGGCAATGA -3'	260 bp for β-actin
β-actin Reverse	5'-TTGATCTTCATGGTGATAGGAGCGAGGGCA -3'	

### Western blotting

Exponentially growing HL-60 cells (2 × 10^6^) were treated with various concentrations of lycorine (0, 1.25, 2.5, 5.0 μM) for 24 h. The cells were then collected and lysed with 100 μl of RIPA buffer (20 mM Tris-HCl, pH 7.5; 150 mM NaCl, 1 mM EDTA, 1% Nonidet P40, 0.5% deoxycholate, 0.1% SDS, 5 mM NaF, and 0.1 mg/ml PMSF), and centrifuged at 13000 rpm at 4°C for 15 min to collect the supernatant. Nuclear proteins were extracted from HL-60 cells by using NE-PER^® ^Nuclear and Cytoplasmic Extraction Reagents according to company procedure (Pierce). Protein concentrations were measured with the BCA protein assay kit (Pierce). Equal amounts of protein (50 μg) from each group were separated by 12% SDS-PAGE and then transferred to a PVDF membrane (Millipore) by blotting on semi-dry electroblotter for 1 h at 4°C. Membranes were blocked in PBS with 0.1% Tween 20 (PBST) containing 5% nonfat dried milk power for 1 h, then incubated 2 h with desired primary antibodies at room temperature. After 3 times washes, the blot was incubated with anti-mouse or anti-rabbit IgG coupled to HRP second antibodies for 1 h at room temperature. After 3 times washes, blots were developed with a chemiluninescene detection kit (ECL; Pierce). The optical density of each band was quantified by densitometric scanning.

### Immunofluorescence staining

Immunofluorescence staining was used to analyze the subcellular distribution of cytochrome c and NF-κB in HL-60 cells induced by lycorine. Cells were collected after treatment with 5.0 μM lycorine or without lycorine for 8 h (for check cytochrome c) or 24 h (for check NF-κB), Cell pellets were smeared on a slide and fixed with 4% formaldehyde for 30 min. Non-specific antigens were blocked with normal serum diluted 1:20 in PBS for 10 min at room temperature. For staining cytochrome c, cells were incubated with the diluted mouse anti-cytochrome c antibody (BioVision) for 2 h and washed 3 times (5 min per wash) with PBS buffer, then incubated with goat anti-mouse Cy3 antibody (Biomeda) for 1 h at room temperature. For staining NF-κB, cells were incubated for 2 h with mouse anti-NF-κB antibody and washed 3 times with PBS buffer, then cells were incubated with biotin-labeled rabbit anti-mouse antibody for 1 h and washed 3 times with PBS buffer. Finally, the cells were treated with streptavidin-biotin complex (SABC)-Cy3 reagent. DNA was stained with 1 μg/ml of 4',6-diamidino-2-phenylindole (DAPI) to locate the nucleus. The images were obtained using confocal laser microscopy (Nikon) with excitation light at a wavelength of 554 nm.

### Statistical analysis

The statistical difference between the groups was determined by AVOVA and Tukey's Studentized Range test. Differences among groups were considered statistically different at *P *< 0.05.

## List of abbreviations

APL: acute promyelogenous leukemia; Bcl2: B cell lymphoma gene 2; Bid: BH3-interacting death domain; Cdc2: cell division cycle 2; Cdk2: cyclin dependent kinase 2; CML: chronic myelogenous leukemia; Cy3: cyanine 3; Mcl-1: myeloid cell leukemia 1; RT-PCR: reverse transcriptase- polymerase chain reaction; SABC: streptavidin-biotin complex; SDS-PAGE: sodium dodecyl sulfate polyacrylamide gel electrophoresis; TGF-β: transforming growth factor-beta; TNF: tumor necrosis factor; TRAIL: tumor necrosis factor-related apoptosis-inducing ligand; TRAF1: TNF receptor-associated factor 1;

## Competing interests

The authors declare that they have no competing interests.

## Authors' contributions

JL performed the immunofluorescence staining, analyzed the data and wrote the manuscript. JLH carried out the experiments of Western blotting, immunofluorescence staining and made the draft of the manuscript as co-first author. BWS carried out the experiments of Western blotting and cell culture. YH contributed to the quantitative analysis of gene expression with real-time PCR. WXH, as the corresponding author, designed the protocol, analyzed the data and revised the manuscript. All authors read and approved the final manuscript.
